# Delta glutamate receptor conductance drives excitation of mouse dorsal raphe neurons

**DOI:** 10.7554/eLife.56054

**Published:** 2020-04-01

**Authors:** Stephanie C Gantz, Khaled Moussawi, Holly S Hake

**Affiliations:** 1 National Institute on Drug Abuse Intramural Research Program, National Institutes of HealthBaltimoreUnited States; 2 Center on Compulsive Behaviors, National Institutes of HealthBethesdaUnited States; 3 Johns Hopkins Medicine, Neurology DepartmentBaltimoreUnited States; Aix-Marseille University, INSERM, INMEDFrance; Oregon Health and Science UniversityUnited States

**Keywords:** delta glutamate receptor, serotonin, noradrenaline, alpha1-adrenergic receptor, leak current, G protein-coupled receptor, Mouse

## Abstract

The dorsal raphe nucleus is the predominant source of central serotonin, where neuronal activity regulates complex emotional behaviors. Action potential firing of serotonin dorsal raphe neurons is driven via α1-adrenergic receptors (α1-A_R_) activation. Despite this crucial role, the ion channels responsible for α1-A_R_-mediated depolarization are unknown. Here, we show in mouse brain slices that α1-A_R_-mediated excitatory synaptic transmission is mediated by the ionotropic glutamate receptor homolog cation channel, delta glutamate receptor 1 (GluD1). GluD1_R_-channels are constitutively active under basal conditions carrying tonic inward current and synaptic activation of α1-A_R_s augments tonic GluD1_R_-channel current. Further, loss of dorsal raphe GluD1_R_-channels produces an anxiogenic phenotype. Thus, GluD1_R_-channels are responsible for α1-A_R_-dependent induction of persistent pacemaker-type firing of dorsal raphe neurons and regulate dorsal raphe-related behavior. Given the widespread distribution of these channels, ion channel function of GluD1_R_ as a regulator of neuronal excitability is proposed to be widespread in the nervous system.

## eLife digest

Serotonin is a chemical that allows cells to communicate in the nervous system of many animals. It is also particularly important in the treatment of mental health disorders: a large number of antidepressants work by preventing nerve cells from clearing away serotonin, therefore increasing the overall level of the molecule in the brain. Yet, exactly how serotonin is released remains unclear.

When a serotonin-producing cell is activated, a series of biochemical processes lead to the creation of an electric current that, ultimately, is required for the cell to release serotonin. This mechanism starts when the α1-adrenergic receptor, a protein at the surface of the cell, detects noradrenaline molecules. However, on its own, the α1-adrenergic receptor is unable to create an electric current: this requires ion channels, another type of protein which can let charged particles in and out of the cell. Here, Gantz et al. set out to determine the identity of the ion channel that allows noradrenaline signals to generate electrical activity in cells which can release serotonin.

Electrical and chemical manipulation of mouse brain slices revealed that an ion channel called delta-glutamate 1 was active in serotonin-producing cells exposed to noradrenaline. In fact, applying toxins that specifically blocked the activity of this channel also prevented the cells from responding electrically to noradrenaline.

Further experiments used mice whose serotonin-producing cells were genetically modified to turn off delta-glutamate 1. In turn, these animals showed anxiety-like behaviors, which could be consistent with a drop in serotonin levels. This is in line with previous human studies showing that patients with depression and other mental health conditions have mutations in the gene for delta-glutamate 1.

Taken together, these results give an insight into the electrical activity of serotonin-producing cells. Further work is now required to examine how changes in the gene that codes for delta-glutamate 1 ultimately affect the release of serotonin. This could potentially help to understand if certain individuals may not be able to properly produce this chemical. As many antidepressants work by retaining serotonin that is already present in the brain, this knowledge could ultimately help patients who do not currently respond to treatment.

## Introduction

Recent reports estimate that 1 in 5 adults worldwide are affected by a mental health disorder, with anxiety and depression being the most common affecting more than 260 million people (GBD 2017 Disease and Injury Incidence and Prevalence Collaborators, 2018). Most current pharmacotherapies to treat these disorders target serotonin receptors or serotonin clearance. The dorsal raphe nucleus is the largest serotonergic nucleus in the brain and the predominant source of central serotonin (5-HT). In vivo, tonic noradrenergic input to the dorsal raphe that activates Gα_q/11_ protein-coupled α1-adrenergic receptors (α1-A_R_s) is required for 5-HT neurons to fire action potentials (Baraban and Aghajanian, 1980; Baraban et al., 1978) and release 5-HT (Clement et al., 1992). In dorsal raphe brain slices, synaptic activation of α1-A_R_s produces a slow membrane depolarization lasting tens of seconds (Yoshimura et al., 1985). Despite having a crucial role in regulating 5-HT neuron excitability, the ion channels responsible for the depolarization remain unknown.

Throughout the central and peripheral nervous system, activation of Gα_q/11_ protein-coupled receptors (G_q_PCRs), namely metabotropic glutamate mGlu_R_s, muscarinic acetylcholine M1 (mACh_R_s), or α1-A_R_s produces slow, noisy inward currents. Multiple mechanisms have been reported to underlie the inward current including: inhibition of K^+^ current (including leak, Ca^2+^-activated, and Kv7/M-current) (Benson et al., 1988; Halliwell and Adams, 1982; Madison et al., 1987; Shen and North, 1992), modulation of TTX-sensitive persistent Na^+^ current (Yamada-Hanff and Bean, 2013), and activation of transient potential receptor canonical (TRPC) (Hartmann et al., 2008; Kim et al., 2003), Na^+^-leak (NALCN) (Lu et al., 2009), or delta glutamate receptor-channels (Ady et al., 2014; Benamer et al., 2018).

The delta glutamate receptors, GluD1_R_ and GluD2_R_, are mysterious members of the ionotropic glutamate receptor family in that they are not gated by glutamate (Araki et al., 1993; Lomeli et al., 1993). One theory is that they are strictly scaffolding proteins or synaptic organizers, rather than ion conducting channels. But wild-type channels have been reported to conduct in response to activation of mGlu_R_s (Ady et al., 2014; Benamer et al., 2018). GluD1_R_ (*Grid1*) mRNA is expressed widely throughout the brain, with notably high levels in the dorsal raphe (Hepp et al., 2015; Konno et al., 2014). Here, we used a combination of in vitro patch-clamp electrophysiology and pharmacology with a CRISPR/Cas9 viral genetic strategy to determine that activation of α1-A_R_s in the dorsal raphe depolarizes neurons via GluD1_R_-channel conductance. We utilize the α1-A_R_-GluD1_R_-EPSC to explore conduction and biophysical properties of GluD1_R_-channels, to ultimately glean a greater understanding of GluD1_R_-channel gating. Lastly, we demonstrate that functional deletion of GluD1_R_-channels in the dorsal raphe produces an anxiogenic behavioral phenotype.

## Results

### Synaptic activation of α1-adrenergic receptors produces an EPSC

**Figure 1.**
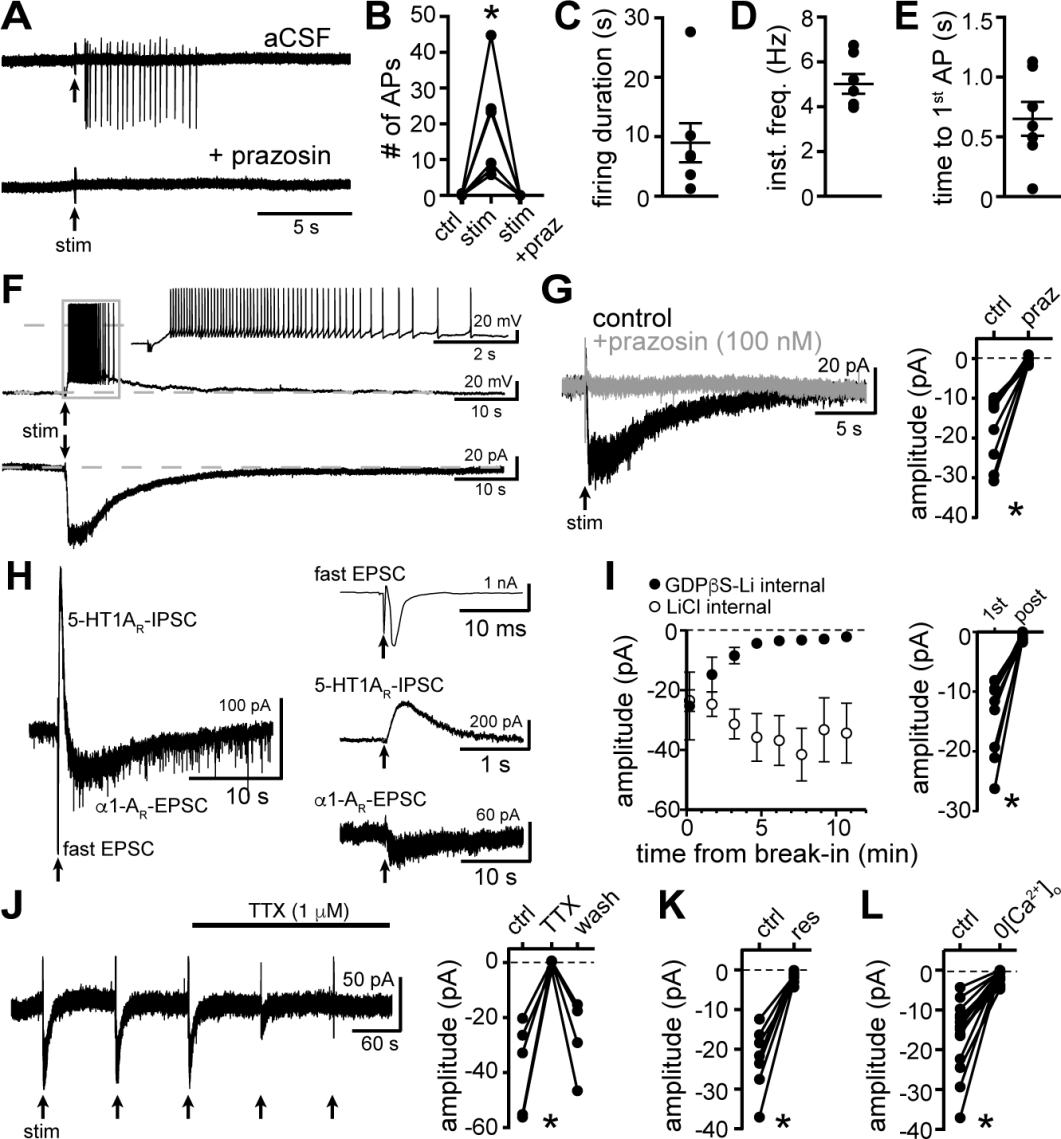
Electrical stimulation evokes long-lasting action potential firing produced by an α1-adrenergic receptor-dependent EPSC. (**A**) Representative traces of cell-attached recording where stimulation of the brain slice (5 stims at 60 Hz) produced action potential firing that was abolished by application of the α1-adrenergic receptor antagonist, prazosin (100 nM). (**B**) Plot of number of action potentials showing the stimulation-induced increase in frequency (p=0.004, n = 6). (**C**) Plot of duration of action potential firing. (**D**) Plot of mean instantaneous frequency of action potential firing over the first 10 s of firing. (**E**) Plot of the latency from stimulation onset to the first action potential. (**F**) Example whole-cell recordings in the same cell, where electrical stimulation of the slice produced prolonged action potential firing in current-clamp (upper trace) and a slow EPSC in voltage-clamp mode (lower trace). (**G**) Bath application of prazosin eliminated the slow EPSC shown in a representative trace (left, baseline adjusted) and in grouped data (right, p=0.002, n = 10). (**H**) Representative traces of a whole-cell recording when the brain slice was stimulated in the absence of antagonists showing the kinetics of the α1-A_R_-EPSC relative to the fast EPSC (peak has been truncated) and 5HT1A_R_-IPSC (left). Subsequent addition of AMPA_R_/Kainate_R_ and GABA-A_R_ and 5-HT1A_R_ antagonists revealed the remaining synaptic current produced by α1-A_R_ activation (right). Time of stimulations are marked by arrows. (**I**) With GDPβS-Li_3_-containing internal solution, the amplitude of the α1-A_R_-EPSC ran down within ~5–20 min of break-in to whole-cell mode; shown in a plot compared with control internal solution containing LiCl only (left) and in grouped data (right, p=0.004, n = 9, 1^st^: first EPSC; post: post-dialysis). (**J**) Bath application of tetrodotoxin (TTX, 1 μM) reversibly eliminated the α1-A_R_-EPSC shown in a representative trace (left, α1-A_R_-EPSC evoked every 90 s (arrows)) and in grouped data (right, p=0.009, n = 4). (**K**) Plot of the inhibition of α1-A_R_-EPSC amplitude by application of reserpine (res, 1 μM, p=0.016, n = 7). (**L**) Plot of the inhibition of α1-A_R_-EPSC amplitude by removal of external Ca^2+^ (0[Ca^2+^]_o_, p=0.0001, n = 14). Line and error bars represent mean ± SEM, * denotes statistical significance. Figure 1—source data 1.Numerical data that were used to generate graphs in Figure 1.

Electrophysiological recordings were made from dorsal raphe neurons in acute brain slices from wild-type mice at 35° C in the presence of NMDA_R_, AMPA_R_, Kainate_R_, GABA-A_R_, and 5-HT1A_R_ antagonists. With cell-attached recordings, a train of 5 electrical stimuli (60 Hz), delivered to the brain slice via a monopolar stimulating electrode, produced firing in previously quiescent neurons, which was blocked by application of the α1-A_R_ antagonist, prazosin (100 nM, Figure 1A). The excitation produced 20±5 action potentials that lasted 9.0±3.0 s, with a latency of 650.6±0.1 ms from onset of stimulation to the first action potential (Figure 1B-E). In whole-cell recording using a potassium-based internal solution, the same train of electrical stimuli produced prolonged action potential firing (Figure 1F). In voltage-clamp mode (V_hold_ -65 mV), the same stimulation produced a slow and long-lasting (27.4±2.3 s, n=10) excitatory postsynaptic current (EPSC, Figure 1F) that was eliminated by the application of prazosin (Figure 1G). Prazosin had no effect on basal whole-cell current (-3.8±3.4 pA, p=0.232, n=10, data not shown) indicating a lack of persistent inward current due to noradrenaline tone. On average, the duration of the α1-A_R_-EPSC was orders of magnitude longer than fast AMPA_R_ channel-mediated EPSCs (~10^3.5^×) and ~18× longer than ‘slow’ 5-HT1A receptor-G protein-coupled inwardly rectifying potassium channel (GIRK)-dependent IPSCs (Gantz et al., 2015a; Figure 1H). To test whether α1-A_R_-EPSCs were dependent on G protein-signaling, recordings were made with an internal solution containing GDPβS-Li_3_ (1.8 mM) in place of GTP. Disruption of G protein signaling with intracellular dialysis of GDPβS-Li_3_ eliminated the α1-A_R_-EPSC within 5-20 mins post-break-in (p=0.004, n=9), whereas dialysis with LiCl alone had no effect on the amplitude of the α1-A_R_-EPSC (p=0.625, n=4, Figure 1I). These findings demonstrate a cell-autonomous requirement of G protein signaling in the generation of the α1-A_R_-EPSC. Application of tetrodotoxin (1 μM) reversibly abolished the α1-A_R_-EPSC, demonstrating a dependence on presynaptic action potentials (Figure 1J). Disruption of the vesicular monoamine transporter with reserpine (1 μM) or removal of external Ca^2+^ also eliminated the α1-A_R_-EPSC, indicating noradrenaline release is vesicular (Figure 1K and L).

### Biophysical properties of the channel

**Figure 2.**
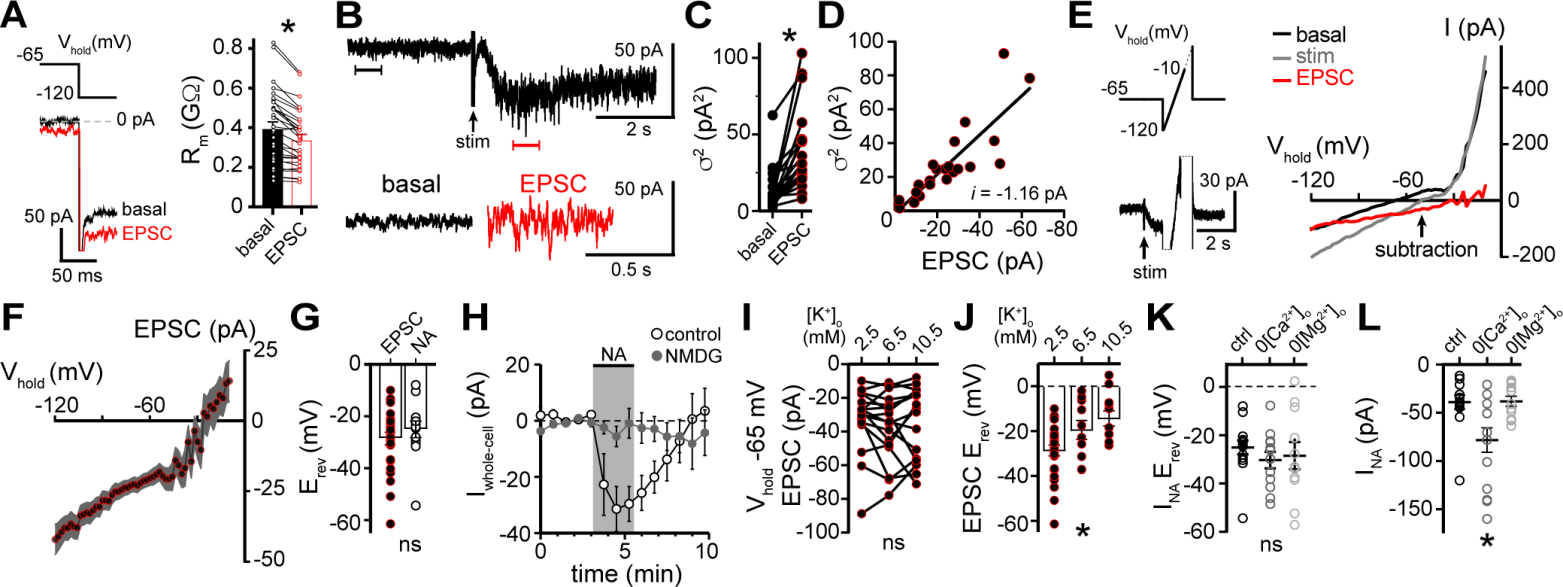
α1-adrenergic receptor-dependent inward current is carried by sodium entry. (**A**) Membrane resistance (R_m_, ΔV −65 to −120 mV) decreased during the α1-A_R_-EPSC indicating an opening of ion channels, as shown in an example trace (left) and in grouped data (right, p<0.0001, n = 31). (**B**) Representative trace of membrane noise during the α1-A_R_-EPSC, brackets denote segments shown below on an expanded scale. (**C**) Membrane noise (variance, σ^2^) increased during the α1-A_R_-EPSC (p<0.0001, n = 22). (**D**) Plot of α1-A_R_-EPSC variance versus mean amplitude, linear fit represents mean unitary current (*i*, r^2^ = 0.713, p<0.0001). (**E**) Slow voltage ramps (1 mV/10 ms, analyzed from −120 to −10 mV) were used to determine the current-voltage relationship of the α1-A_R_-EPSC (subtraction), determined by subtracting current at the peak of the α1-A_R_-EPSC (stim) from current measured in control conditions just prior to stimulation (basal). Current generated during ramps were truncated for clarity. (**F**) Current-voltage relationship of the α1-A_R_-EPSC from grouped data. Shaded area represents mean ± SEM. (**G**) Plot of reversal potentials (E_rev_) of the α1-A_R_-EPSC and I_NA_ (p>0.999, n = 26 and 14). (**H**) Replacing 126 mM NaCl with NMDG eliminated inward I_NA_, shown in a time-course plot (V_hold_−65 mV, p<0.0001, n = 14 and 13). (**I**) Plot of α1-A_R_-EPSC amplitudes measured at V_hold_−65 mV, in 2.5, 6.5, and 10.5 mM [K^+^]_o_ (p=0.162, n = 17). (**J**) Plot of α1-A_R_-EPSC reversal potential (E_rev_) with varying concentration of external K^+^ ([K^+^]_o_), demonstrating a depolarizing shift in E_rev_ as external K^+^ was increased (p=0.010, n = 26, 10, and 11). (**K**) Plot of reversal potentials (E_rev_) of I_NA_, demonstrating no significant difference between control conditions (ctrl), and after removal of external Ca^2+^ (0[Ca^2+^]_o_, p=0.49, n = 14 and 12) or Mg^2+^ (0[Mg^2+^]_o_, p=0.73, n = 14 and 11). (**L**) Plot of the amplitude of I_NA_ (V_hold_−65 mV) demonstrating an augmented I_NA_ amplitude in 0[Ca^2+^]_o_ (p=0.017, n = 14), but not in 0[Mg^2+^]_o_, (p>0.9999, n = 11) as compared with control conditions (n = 14). Line and error bars represent mean ± SEM, * denotes statistical significance, ns denotes not significant. Figure 2—source data 1.Numerical data that were used to generate graphs in Figure 2.

Under our recording conditions, resistance of the membrane (R_m_) significantly decreased during the α1-A_R_-EPSC, indicative of opening of ion channels (Figure 2A). Membrane noise variance (σ^2^) increased significantly during the EPSC compared to membrane noise under basal conditions (Figure 2B and C). The α1-A_R_-EPSC σ^2^ – amplitude relationship was well fit by linear regression, suggestive of a consistent conductance state, yielding an estimate of a -1.16 pA unitary current (Figure 2D). Voltage ramps from -120 to -10 mV (1 mV/10 ms) before and during the α1-A_R_-EPSC (Figure 2E) showed that the current reversed polarity at -28.6±2.4 mV (Figure 2E-G). Exogenous application of noradrenaline (30 μM, in the presence of α2-A_R_ antagonist, idazoxan, 1 μM) produced an inward current (I_NA_) with a similar reversal potential (-25.1±2.9 mV, Figure 2G). Replacing extracellular Na^+^ (126 mM) with N-methyl D-glucamine (NMDG) completely abolished inward I_NA_, suggesting Na^+^ as the prominent charge carrier (Figure 2H). Increasing extracellular K^+^ from 2.5 to 6.5 or 10.5 mM, expected to shift E_k_ from -107 to -81 and -69 mV, respectively, had no effect on the amplitude of the α1-A_R_-EPSC at V_hold_ -65 mV (Figure 2I) nor -120 mV (p=0.692, n=11, data not shown), but produced a significant depolarizing shift in E_rev_ of the α1-A_R_-EPSC (Figure 2J), suggesting the channel is also permeable to K^+^, and may be 2-3× as permeable to K^+^ as Na^+^. Removal of external MgCl_2_ had no significant effect on E_rev_ (-28.5±5.7 mV), nor on the amplitude of I_NA_ (Figure 2K and L). Removal of external CaCl_2_ also had no effect on E_rev_ (-30.3±3.5 mV) but significantly augmented inward I_NA_, (Figure 2K and L). Taken together, the data suggest that α1-A_R_-dependent current, whether produced by vesicular release of noradrenaline or exogenous noradrenaline application is carried through a mixed cation channel, with inward current carried predominantly by Na^+^ entry. Here, measurements of E_rev_ assume voltage-independence of the channel and the signaling mechanism by which α1-A_R_ signal to the channel. To test for voltage-dependence, we employed a two-pulse voltage-step protocol. Current was measured at V_hold_ -120 mV following a conditioning pre-pulse (-120 to 30 mV, 150 ms) before and after application of noradrenaline (Figure 2—figure supplement 1A and B). I_NA_ was isolated by subtracting the current under basal conditions from the current during noradrenaline. Conductance (G_NA_) was calculated, using an E_rev_ of -25.1 mV. Conditioning depolarizing pre-pulses incrementally reduced G_NA_ and the increase in membrane noise induced by noradrenaline measured at V_hold_ -120 mV (Figure 2—figure supplement 1C and D), demonstrating voltage-dependence of inward I_NA_, such that depolarization reduced conductance.

**Figure 2—figure supplement 1.**
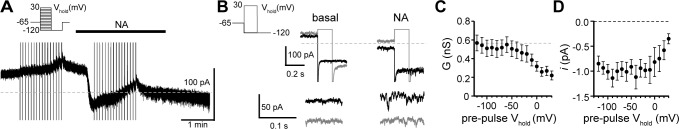
Tail current analysis reveals voltage-dependence of the α1-adrenergic receptor-dependent inward current. (**A**) I_NA_ was isolated using a two-pulse voltage protocol by subtracting current measured during voltage steps (shown in left inset) at the peak of inward current from noradrenaline application from current measured in control conditions just prior to noradrenaline application. (**B**) Representative traces of current at V_hold_−120 mV following conditioning pre-pulses to −120 mV (black) or to 30 mV (gray) in control conditions (basal) and after noradrenaline. Traces below show membrane noise at V_hold_−120 mV on an expanded scale. (**C**) Conductance (G_NA_) was measured at peak I_NA_ from V_hold_−120 mV, calculated using a E_rev_ of −25.1 mV. Plot of G_NA_ versus pre-pulse V_hold_, demonstrating a decrease in G_NA_ with depolarizing pre-pulses (linear trend of −0.022 nS/mV, p<0.0001). (**D**) Plot of unitary current (*i*, measured at V_hold_−120 mV) versus pre-pulse V_hold_ demonstrating a decrease in *i* with depolarizing pre-pulses (linear trend of 0.026 pA/mV, p=0.002). Line and error bars represent mean ± SEM, * denotes statistical significance.

### α1-adrenergic receptors modulate tonic GluD1_R_-channel current

**Figure 3.**
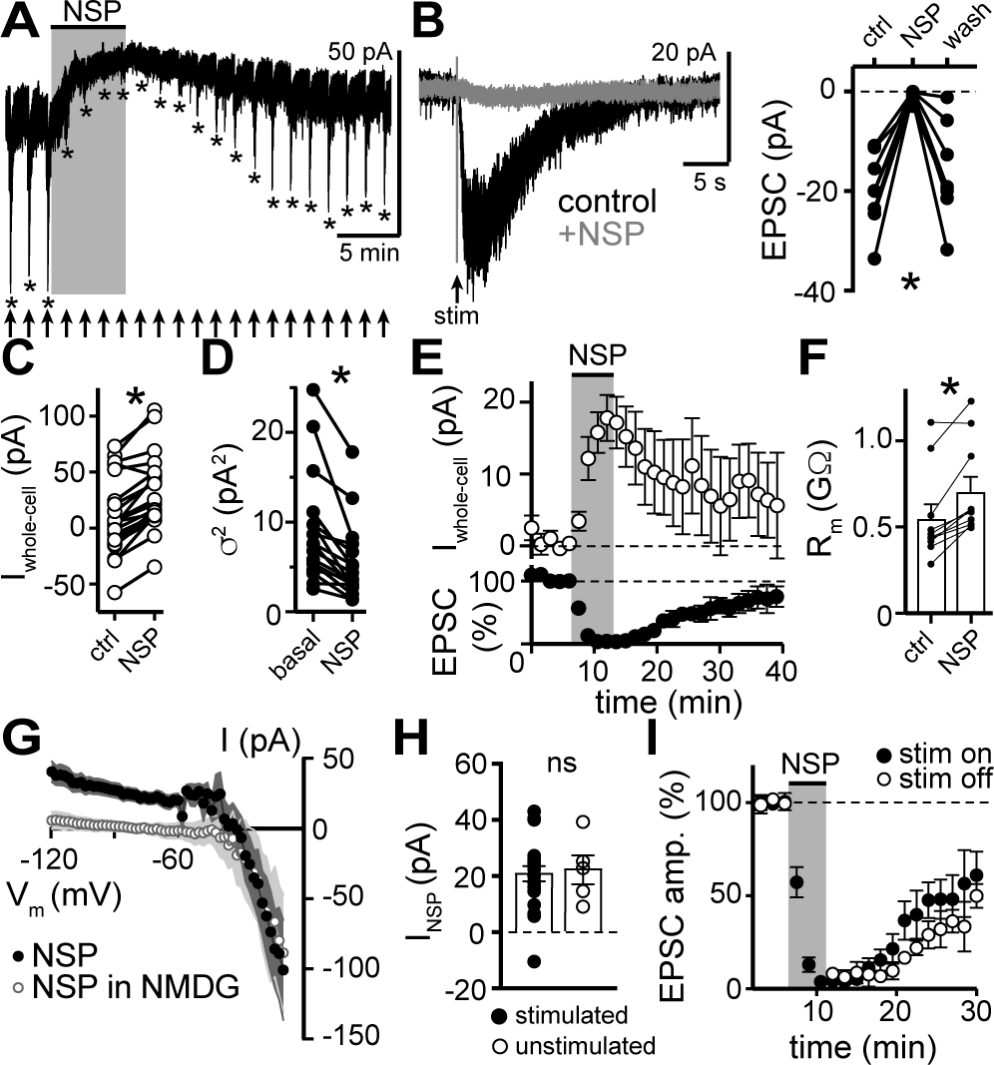
NASPM blocks the α1-A_R_-EPSC and a tonic sodium inward current. (**A**) Example whole-cell voltage-clamp recording of the basal whole-cell current and the α1-A_R_-EPSC evoked every 90 s prior to, during, and after bath application of NASPM (NSP, 100 μM). Time of stimulations are marked by arrows and peak of the α1-A_R_-EPSC are marked by asterisks. (**B**) NASPM completely eliminated the α1-A_R_-EPSC shown in representative traces (left, baseline adjusted) and in grouped data (right, p=0.001, n = 8). (**C**) Bath application of NASPM produced an apparent outward current (p<0.0001, n = 21). (**D**) Membrane noise (variance, σ^2^) decreased following NASPM (NSP, p<0.0001, n = 19).(**E**) Time course of the inhibition of the α1-A_R_-EPSC amplitude (bottom) and apparent outward current (top) by application of NASPM. (**F**) Membrane resistance (R_m_, ΔV −65 to −75 mV) increased during bath application of NASPM, indicating the apparent outward current was due to ion channels closing (p=0.004, n = 10). (**G**) Current-voltage relationship of apparent outward current produced by NASPM (n = 8). Replacing 126 mM NaCl eliminated the apparent outward current (n = 11), suggesting a block of a tonic inward Na^+^ current. Shaded area represents mean ± SEM. (**H**) Plot of amplitude of NASPM-induced apparent outward current in stimulated and unstimulated brain slices demonstrating no effect of prior electrical stimulation (p=0.850, n = 21 and 5). (**I**) Time course of the inhibition of the α1-A_R_-EPSC amplitude by application of NASPM, demonstrating identical block of the α1-A_R_-EPSC whether or not α1-A_R_-EPSCs were evoked during NASPM application. Line and error bars represent mean ± SEM, ns indicates not significant, * denotes statistical significance. Figure 3—source data 1.Numerical data that were used to generate graphs in Figure 3.

To assess involvement of GluD1_R_-channels in carrying the α1-A_R_-EPSC, we applied 1-Naphthyl acetyl spermine (NASPM), a synthetic analogue of Joro spider toxin that is an open-channel blocker of some other Ca^2+^-permeable ionotropic glutamate receptors (Blaschke et al., 1993; Guzmán et al., 2017; Koike et al., 1997) and of GluD_R_-channels (Benamer et al., 2018; Kohda et al., 2000). Application of NASPM (100 μM, 6 min) blocked the α1-A_R_-EPSC (96.0 ± 12.5% reduction), which recovered to baseline after a wash of >30 mins (Figures 3A, B, E and I). NASPM also produced an apparent outward current (I_NSP_) of 20.5 ± 3.7 pA with an E_rev_ of −31.4 ± 4.8 mV (Figures 3A, C, E and G) and a reduction in membrane noise (Figure 3A and D). After washout, I_NSP_ reversed with a similar time course of recovery of the α1-A_R_-EPSC (Figure 3E). I_NSP_ was associated with an increase in R_m_ (Figure 3F) indicating a closure of channels. Replacing extracellular Na^+^ (126 mM) with NMDG eliminated I_NSP_ (Figure 3G). Thus, I_NSP_ was due to block of tonic Na^+^-dependent inward current. I_NSP_ was not dependent on prior electrical stimulation of the brain slice, as the magnitude of I_NSP_ was similar between stimulated and unstimulated brain slices (Figure 3H). Given that NASPM is an open-channel blocker (Koike et al., 1997), we tested whether electrically evoking an α1-A_R_-EPSC during the application of NASPM was required for block. After obtaining a steady α1-A_R_-EPSC baseline, NASPM was applied for 6 min without stimulating the brain slice. The α1-A_R_-EPSC was blocked when stimulation was reapplied (Figure 3I), indicating that the channels underlying the α1-A_R_-EPSC were already blocked. Thus, the α1-A_R_-EPSC is mediated by channels that are at least transiently open at rest and may be the same channels underlie the apparent outward current induced by NASPM.

**Figure 4.**
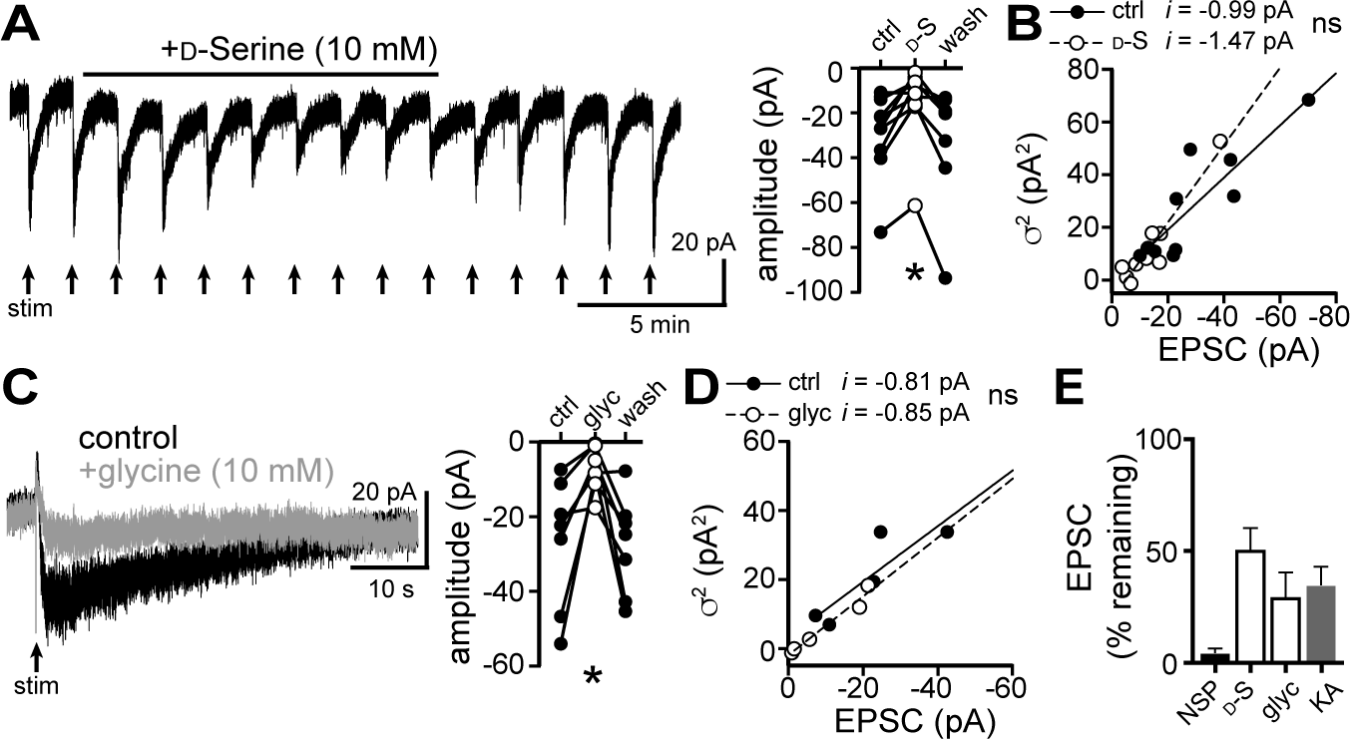
D-serine and glycine reduce the α1-A_R_-EPSC. (**A**) Bath application of D-serine (10 mM) reversibly reduced the α1-A_R_-EPSC, shown in a representative trace (left) and in grouped data (right, p=0.001, n = 7). (**B**) Plot of α1-A_R_-EPSC variance versus mean amplitude prior to (ctrl) and after reduction by D-serine (D–S), linear fit represents mean unitary current (**i**), demonstrating no change in *i* with D-serine (p=0.165, n = 10 and 10). (**C**) Bath application of glycine (10 mM) reversibly reduced the α1-A_R_-EPSC, shown in representative traces (left, baseline adjusted) and in grouped data (right, p=0.015, n = 7). (**D**) Plot of α1-A_R_-EPSC variance versus mean amplitude prior to (ctrl) and after reduction by glycine (glyc), linear fit represents mean unitary current (**i**), demonstrating no change in *i* with glycine (p=0.895, n = 5 and 5). (**E**) Summarized data of percent remaining in α1-A_R_-EPSC after NASPM (NSP, 100 μM), D-serine (D-S, 10 mM), glycine (glyc, 10 mM), or kynurenic acid (KA, 1 mM). Line and error bars represent mean ± SEM, ns indicates not significant, * denotes statistical significance. Figure 4—source data 1.Numerical data that were used to generate graphs in Figure 4.

GluD_R_s bind the amino acids D-serine and glycine, both of which partially reduce constitutively open mutant and wild-type GluD_R_ channel current (Ady et al., 2014; Benamer et al., 2018; Naur et al., 2007; Yadav et al., 2011), likely by inducing a conformational change in the channel that resembles a desensitized state (Hansen et al., 2009). Application of D-serine (10 mM, 13.5 min) reduced the amplitude of the α1-A_R_-EPSC by 49.7 ± 9.6% (Figure 4A and E), without affecting unitary channel current (Figure 4B). Application of glycine (10 mM, 4.5 mins, in the presence of the glycine receptor antagonist, strychnine (10 μM), also reduced the amplitude of the α1-A_R_-EPSC by 70.9 ± 11.0% (Figure 4C and E), without affecting unitary channel current (Figure 4D). Lastly, we found that application of the glutamate receptor antagonist kynurenic acid (1 mM, 10.5 min) reduced the α1-A_R_-EPSC amplitude by 65.6 ± 8.3% (Figure 4E).

**Figure 5.**
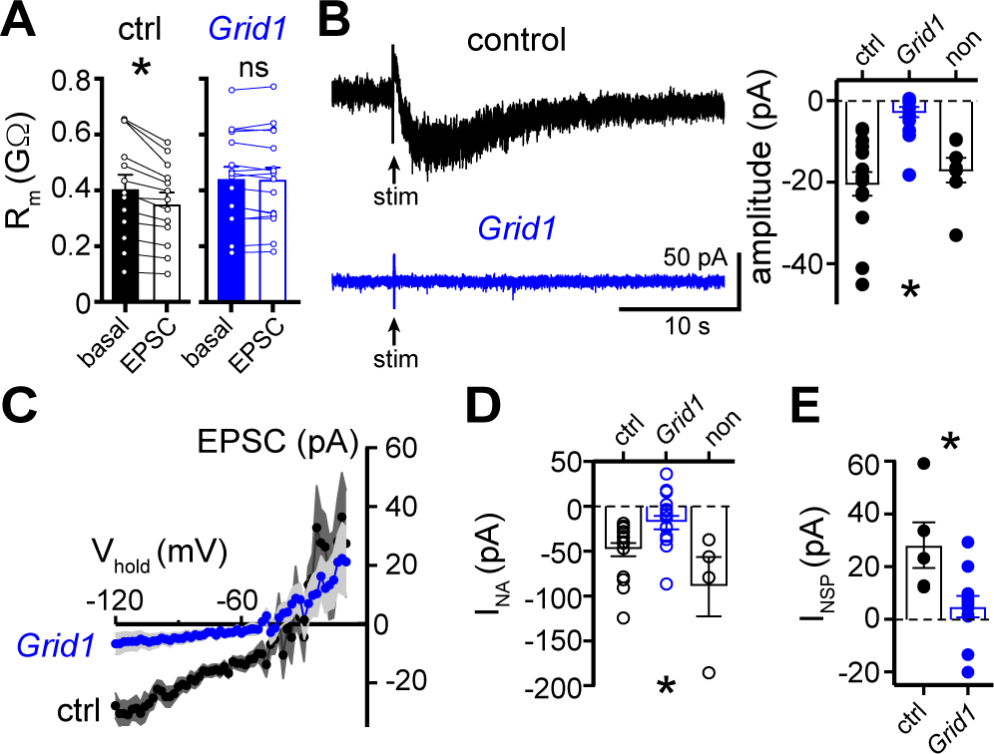
The α1-A_R_-EPSC is eliminated by targeting of GluD1_R_-channels via CRISPR/Cas9. (**A**) Membrane resistance (R_m_, ΔV −65 to −120 mV) decreased after stimulation in transduced neurons from mice injected with AAV-Cas9 and AAV-empty (ctrl, p=0.0002, n = 13), but not in transduced neurons from mice injected with AAV-Cas9 and AAV-*Grid1* (*Grid1*, p=0.562, n = 16). (**B**) Representative traces (left) and grouped data (right, p<0.0001, n = 15 and 16 and 7) demonstrating the presence of an α1-A_R_-EPSC in transduced neurons from control mice, but not from *Grid1* mice. Neighboring non-transduced neurons from mice injected with AAV-Cas9 and AAV-*Grid1* (non) had an α1-A_R_-EPSC that was indistinguishable from transduced neurons from control mice (p>0.999). (**C**) Current-voltage relationship of the α1-A_R_-EPSC from control (n = 13) and *Grid1* (n = 16) grouped data. Shaded area represents mean ± SEM. (**D**) Targeting GluD1_R_ reduced the inward current to noradrenaline (NA, I_NA,_30 μM) as compared to transduced neurons from control mice and neighboring non-transduced neurons (p=0.004, n = 16 and 16 and 4). Inward I_NA_ in non-transduced neurons from mice injected with AAV-Cas9 and AAV-*Grid1* was similar to transduced neurons from control mice (p=0.631). (**E**) Targeting GluD1_R_ reduced the tonic inward current revealed by bath application of NASPM (100 μM, I_NSP_) as compared to transduced neurons from control mice (p=0.009, n = 5 and 11). Line and error bars represent mean ± SEM, * denotes statistical significance, ns denotes not significant. Figure 5—source data 1.Numerical data that were used to generate graphs in Figure 5.

Next, a viral genetic strategy was used to functionally delete GluD1_R-_channels by targeting the encoding gene, *Grid1*, via CRISPR/Cas9 (Figure 5—figure supplement 1A–C). In brief, one of two cocktails of AAV1 viruses were microinjected into the dorsal raphe of wild-type mice. The *Grid1* cocktail that targeted GluD1_R_-channels included AAV1 viruses encoding Cas9, and mouse *Grid1* guide RNA with a nuclear envelope-embedded enhanced green fluorescent protein (eGFP) reporter. A separate cohort received a control cocktail of AAV1 viruses encoding Cas9 and eGFP reporter (control). Brain slices were prepared after >4 weeks and the dorsal raphe was microdissected and frozen on dry ice to assess the mutation of *Grid1*. Restriction enzyme site-digested PCR confirmed in vivo mutation of *Grid1* at the expected site (Figure 5—figure supplement 1D). In separate *Grid1* and control cohorts, brain slices were prepared and whole-cell voltage-clamp recordings were made from transduced and non-transduced neurons visualized in brain slices by expression of eGFP. In eGFP^+^ neurons from control mice, electrical stimulation produced a decrease in R_m_ and an α1-A_R_-EPSC, and bath application of noradrenaline caused inward I_NA_ (Figure 5). However, in eGFP^+^ neurons from *Grid1* mice, electrical stimulation did not change R_m_ (Figure 5A) and no α1-A_R_-EPSC was detected above baseline noise (Figure 5B and C). In addition, inward I_NA_ was substantially smaller in eGFP^+^ neurons from *Grid1* mice, as compared to eGFP^+^ neurons from control mice (Figure 5D). In the same slices from *Grid1* mice, eGFP^-^ neurons still had an α1-A_R_-EPSC and inward I_NA_ (Figure 5B and D). Lastly, bath application of NASPM produced an apparent outward current in eGFP^+^ neurons from control mice, but not from *Grid1* mice (Figure 5E). Taken together, these results demonstrate that conduction through GluD1_R_-channels is necessary for the α1-A_R_-EPSC and the NASPM-sensitive tonic inward current.

**Figure 5—figure supplement 1.**
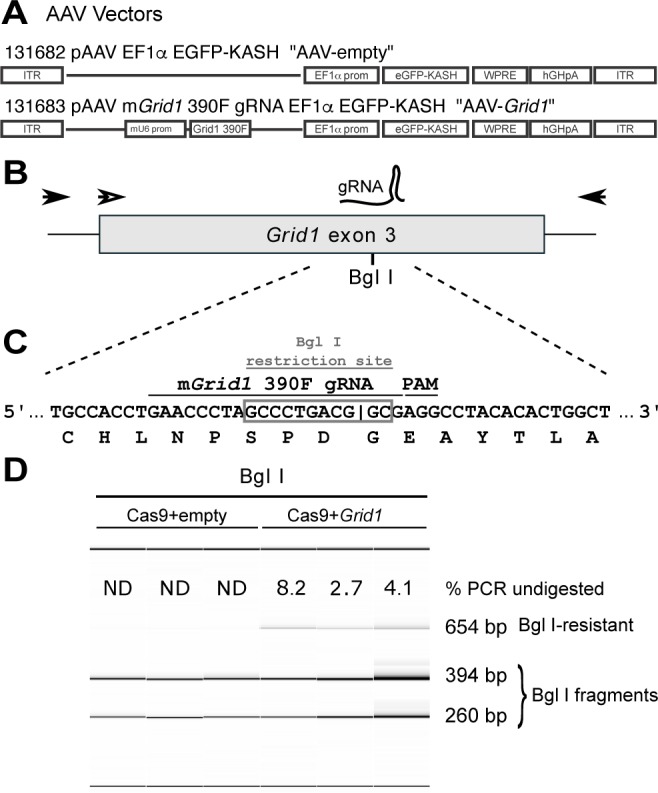
Design and testing of guide RNA targeting mouse *Grid1*. (**A**) A graphic representation of AAV vectors used in conjunction with AAV-Cas9 to encode eGFP reporter only (top, AAV-empty) and mouse *Grid1* guide RNA with eGFP reporter (bottom, AAV-*Grid1*). (**B**) A graphic representation of the third exon of mouse *Grid1*, marked with the location of the gRNA (390F) and the overlapping Bgl I restriction site. Filled arrows represent the location of primers used for PCR amplification while the hollow arrow indicates the primer used for sequencing. (**C**) A nucleotide representation of gRNA target site with the translated peptide sequence underneath. The Bgl I recognition site (GCCNNNN^NGGC) is contained by the grey box and overlaps with the predicted Cas9 cleavage site (|). (**D**) Fragment analysis on Bgl I-digested *Grid1* PCR products amplified from the dorsal raphe of mice injected with (AAV-Cas9 and AAV-empty) or (AAV-Cas9 and AAV-*Grid1*). ‘% PCR undigested’ refers to the amount of the undigested PCR product relative to the total PCR product as determined by AATI fragment analysis.

### Functional deletion of GluD1_R_-channels in the dorsal raphe produces a behavioral phenotype

**Figure 6.**
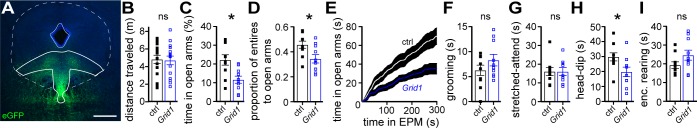
Loss of functional GluD1 receptors in the dorsal raphe produces an anxiogenic behavioral phenotype in mice. (**A**) Example maximum intensity projection confocal image of spread of viral transduction following dorsal raphe microinjection of AAV-Cas9 and AAV-*Grid1* using an eGFP reporter; scale bars, 0.5 mm. Image was registered and aligned with plate 69 (Franklin and Paxinos mouse brain atlas) with dorsal raphe outlined in solid white. (**B**) Plot of distance traveled in 30 mins in a dark arena, demonstrating no difference in horizontal locomotion between mice with dorsal raphe microinjections with AAV-Cas9 and AAV-empty (ctrl) versus with AAV-Cas9 and AAV-*Grid1* (p=0.762, n = 18 and 15). (**C**) In an elevated plus maze, *Grid1* mice spent less time in the open arms as compared with control-transduced mice (p=0.007, n = 10 and 10). (**D**) *Grid1* mice made proportionally fewer entries to the open arms compared to control-transduced mice (p=0.033, n = 10 and 10). (**E**) Plot of cumulative time spent in the open arms of an elevated plus maze (EPM). (**F**) Plot of time spent grooming, demonstrating no different between control-transduced and *Grid1* mice (p=0.481, n = 10 and 10). (**G**) Plot of time spent in stretched-attend posture, demonstrating no difference between control-transduced and *Grid1* mice (p=0.968, n = 10 and 9). (**H**) *Grid1* mice spent less time looking over the edge of the open arms (head-dip) than control-transduced mice (p=0.018, n = 10 and 10). (**I**) Plot of time spent rearing in the enclosed arms, demonstrating no difference between control-transduced and *Grid1* mice (p=0.143, n = 10 and 10). Line and error bars represent mean ± SEM, n = number of mice, * denotes statistical significance, ns denotes not significant. Figure 6—source data 1.Numerical data that were used to generate graphs in Figure 6.

To assay a functional role of GluD1_R_-channels in dorsal raphe-related behavior, wild-type mice received a microinjection into the dorsal raphe of either *Grid1* or control virus cocktails. Behavioral assays were conducted >4 weeks post-injection, then the accuracy of the dorsal raphe injection and limited-spread of transduction was verified post-hoc by immunohistochemistry (Figure 6A). Basal locomotion was assayed in a dark arena. There was no difference between the two groups in the total distance traveled (Figure 6B) nor in the velocity of movements between control and *Grid1* mice (p=0.772, n = 18 and 16, data not shown). Next, mice were tested on an elevated plus maze in a well-lit room, an experimental assay of rodent anxiety behavior (Walf and Frye, 2007) known to involve both serotonergic and non-serotonergic neurons in the dorsal raphe (Lawther et al., 2015). *Grid1* mice spent less time in the open arms when compared to control mice (Figure 6C–E). Control and *Grid1* mice made a similar total number of entries to either open or enclosed arms (control: 39.4 ± 2.0; *Grid1*: 36.0 ± 2.3, p=0.697), but *Grid1* mice made proportionally fewer entries to the open arms (Figure 6D). Time spent grooming or in stretched-attend postures were similar between control and *Grid1* mice (Figure 6F and G). Since movement in the elevated plus maze reflects conflict between innate drive to explore of a novel environment and natural avoidance of open spaces (Walf and Frye, 2007), we also examined exploratory behaviors. *Grid1* mice spent less time lowering their head over the edge of the open arms than control mice (head-dipping, Figure 6H), suggestive of decreased exploratory behavior. However, *Grid1* mice spent a similar amount of time rearing in the enclosed arms compared to control mice (Figure 6I) suggesting innate exploratory drive in the enclosed arms was intact. Taken together, these results are indicative of heightened anxiety after functional deletion of GluD1_R_-channels in the dorsal raphe.

## Discussion

### Physiological relevance of GluD1_R_-channels to dorsal raphe function

In vivo, 5-HT neurons in the dorsal raphe require noradrenaline release and subsequent activation of α1-A_R_s to maintain persistent action potential firing (Baraban and Aghajanian, 1980). The activation of α1-A_R_s in the dorsal raphe by exogenous agonist was thought to depolarize neurons through net reduction of K^+^ conductance, transiently activating calcium-activated K^+^ current while persistently decreasing another K^+^ current, and by activation of an unidentified non-K^+^ conductance (Pan et al., 1994). In a more recent study in the dorsal raphe, Brown et al. (2002) reported that activation of α1-A_R_s, induces Na^+^-dependent inward current with an E_rev_ of −23 mV, similar to our findings. Our study identifies GluD1 receptor-channels as the ion channel that carries this mixed cation current, indicating that modulation of GluD1_R_-channels is a key constituent in driving persistent action potential firing of the 5-HT neurons. In principle, inward GluD1_R_-channel current may bring the membrane potential to threshold, but recruitment of other voltage-gated ion channels is expected to underlie the persistent pacemaker-like activity. Intriguingly, Brown et al. (2002) demonstrated that activation of G_q_ protein-coupled histamine H_1_ and orexin OX_2_ receptors also produced an inward current that was occluded by the α1-A_R_-dependent current. Whether these receptors, and other G_q_PCRs, augment GluD1_R_-channel current remains to be determined.

Dysregulation of the 5-HT signaling neuropsychiatric disorders is well-established. Pharmacotherapies to boost serotonin signaling are common and often efficacious in some of these conditions. Genetic association studies have identified *GRID1* as a susceptibility gene for psychiatric conditions, including schizophrenia, major depressive disorder, bipolar disorder, autism spectrum disorder, and alcohol dependence (Edwards et al., 2012; Fallin et al., 2005; Griswold et al., 2012). Global *Grid1* knock-out mice display abnormal social behaviors, including heightened aggression and decreased social interaction, as well as altered emotional behaviors (Yadav et al., 2012) that are analogous to features of neuropsychiatric conditions in humans. Our study found that functional deletion of GluD1_R_-channels, specifically in the dorsal raphe, produces a heightened anxiety-like response in the elevated plus maze without changing basal locomotion and exploratory behaviors in non-threatening environments. Previous studies have demonstrated that both 5-HT and non-5-HT/GABAergic dorsal raphe neurons are activated by aversive, anxiety or fear-producing stimuli (Seo et al., 2019; Silveira et al., 1993), with regional subpopulation specificity (Grahn et al., 1999; Grahn et al., 2019; Lawther et al., 2015). Our viral strategy functionally deleted GluD1_R_-channels in a non-specific manner, targeting all dorsal raphe neurons, including 5-HT and GABAergic neurons. Given the rich diversity of dorsal raphe neuron subtypes and subdivisions within the 5-HT neurons (Huang et al., 2019; Luo et al., 2015; Ren et al., 2018), future work will be needed to parse the behavioral role of GluD1_R_-channels with subnuclei/subpopulation specificity.

### Metabotropic-ionotropic receptor crosstalk modulates ion channel function of GluD1_R_

GluD_R_ have been characterized as scaffold proteins or synaptic organizers, regulating LTD, endocytosis and trafficking of AMPA_R_, formation of excitatory and inhibitory synapses, and spine density, independent of ion conduction through the pore (Fossati et al., 2019; Hirai et al., 2003; Schmid and Hollmann, 2008; Tao et al., 2018). Similarly, NMDA_R_ are known to signal through non-ionotropic or ‘metabotropic’ mechanisms where ion conduction is not required, to regulate LTD, AMPA_R_ endocytosis, and spine morphology (Dore et al., 2016). The ability of GluD_R_-channels to carry ionic current does not conflict with its known role as a synaptic organizer, but rather expands the similarities between NMDA_R_ and GluD_R_.

The largest obstacle in advancing the understanding of the ionotropic nature of GluD_R_ is the lack of known agonist and inability to gate the intact channel. The majority of studies have been performed on constitutively open mutant or chimeric channels. In domain-swapped chimeric channels, agonist binding to the ligand-binding domain (LBD) of AMPA_R_ or Kainate_R_ opens the GluD_R_-channel pore and generates a substantial current, but the LBD of GluD_R_ on the pore region of AMPA_R_ or Kainate_R_-channels fails to generate current (Orth et al., 2013; Schmid et al., 2009). Two prior studies have demonstrated that in heterologous systems and brain slices, activation of metabotropic glutamate receptors (mGlu_R_) produces an inward current carried by GluD1_R_- (Benamer et al., 2018) or GluD2_R_-channels (Ady et al., 2014), concluding that mGlu_R_ activation triggers gating of GluD_R_ channels. The congruous explanation of our results is that, in dorsal raphe neurons, GluD1_R_-channels are functional and open under basal conditions, carrying subthreshold, tonic Na^+^ current. Activation of α1-A_R_s, by exogenous agonist or synaptic release of noradrenaline modulates gating of GluD1_R_-channels and excites dorsal raphe neurons by increasing tonic GluD1_R_-channel inward current.

In general, the kinetics of iGlu_R_ synaptic currents are controlled by the lifetime of the receptor-agonist complex and the rate of desensitization and deactivation. The presence of ambient levels of glutamate and glycine along with slow desensitization activate NMDA_R_ to produce a tonic inward current (Sah et al., 1989). Our results demonstrate that GluD1_R_ are functional ion channels, but whether they function as ligand-gated receptor-channels that open in response to a chemical signal, is not yet determined. What remains to be understood are the conditions that permit GluD1_R_-channel opening and why their activation has been largely elusive in heterologous expression systems. Reminiscent of times before the discovery of glycine as a necessary co-agonist at NMDA_R_ (Johnson and Ascher, 1987; Kleckner and Dingledine, 1988), it may be that an endogenous agonist needed for gating is present in brain slices. Alternatively, it is possible GluD1_R_-channels are gated by an intracellular factor or require expression of accessory or interacting protein (Tomita, 2010). Tonic activation of α1-A_R_s cannot explain the tonic inward current as α1-A_R_ antagonism did not change basal whole-cell current.

The mechanism by which α1-A_R_s increase GluD1_R_-channel current also remains to be described and may be distinct from the tonic activation. It is well-established that G_q_PCRs, especially mGlu_R_ and mACh_R,_ bidirectionally change NMDA_R_ and AMPA_R_ ionic currents, producing the two major forms of synaptic plasticity, long-term potentiation (LTP) and long-term depression (LTD), in part through a variety of distinct postsynaptic mechanisms (Hunt and Castillo, 2012). To our knowledge, the duration of the α1-A_R_-EPSC (~27 s) is exceptional for any known synaptic current and more closely resembles the duration of short-term synaptic plasticity; for instance, endocannabinoid-mediated short-term depression (Lu and Mackie, 2016). Canonically, G_q_PCRs activate phospholipase C which hydrolyzes the integral membrane lipid phosphatidylinositol 4,5-bisphosphate (PIP2) into inositol triphosphate (IP_3_) and diacylglycerol. PIP2 stabilizes Kv7 channels such that PIP2 hydrolysis following mACh_R_ activation accounts for inhibition of M-current (Suh and Hille, 2002). In contrast, PIP2 inhibits TRPV4 channels, such that G_q_PCR-dependent PIP2 depletion allows for TRPV4 channels to open (Harraz et al., 2018). By the same signaling cascade, G_q_PCRs stimulate the production of the endocannabinoid, 2-AG, that can act directly on ion channels in the membrane (Gantz and Bean, 2017). Thus, one possibility is that α1-A_R_s modulate GluD1_R_-channels through membrane lipid signaling, involving PIP2, diacylglycerol, or 2-AG, as it can take tens-of-seconds to minutes for ion channels to recover from modulation by membrane lipids (Gantz and Bean, 2017; Suh and Hille, 2002). Alternatively, there may be direct modulation of GluD1_R_-channels by G protein subunits or activation of protein kinase signaling cascades. The inclusion of the calcium-chelator BAPTA in the internal recording solution makes it unlikely that α1-A_R_s modulate GluD1_R_-channels via IP_3_ and calcium release from intracellular stores (Hoesch et al., 2004). Largely, it remains to be seen whether these intracellular signaling cascades, many of which are known to affect NMDA_R_- and AMPA_R_-channels, modify GluD_R_-channels.

In heterologous systems and constitutively open mutant GluD_R_-channels, the current reverses polarity around 0 mV (Zuo et al., 1997), akin to AMPA_R_- and NMDAR-channels, while our results show E_rev_ of ~ −30 mV. While slow voltage-ramps were employed to minimize space-clamp error, we cannot rule out that some of the difference may be attributed to space-clamp error in brain slices, especially since the magnitude of subtracted current is small relative to total membrane current at depolarized potentials. However, there are many reports of inward currents produced by activation of many different G_q_PCRs with reversal potentials between −40 and −23 mV (Awad et al., 2000; Brown et al., 2002; Yamada-Hanff and Bean, 2013) under different recording conditions; a commonality that is unlikely to be accounted for by space-clamp error alone. Tail current analysis revealed voltage-dependence of I_NA_, such that depolarization reduced conductance. These data may reflect block of GluD1_R_-channels by endogenous intracellular polyamines, as established for calcium-permeable AMPA_R_- and Kainate_R_-channels (Bowie and Mayer, 1995). Another important consideration is that our measurements may be subject to voltage-dependence of the signaling pathway between α1-A_R_s and GluD1_R_-channels. Taken together, measurements here should be considered an estimate of GluD1_R_-channels, and more precisely as the current-voltage relationship of the α1-A_R_s-GluD1_R_-channel signaling complex.

### Summary

In summary, the α1-A_R_-mediated depolarization of dorsal raphe neurons that drives action potential firing in vivo is carried by the mixed cation channel, GluD1_R_. Thus in addition to their role as a scaffold protein, GluD1_R_ are functional ion channels that critically regulate neuronal excitability. Many of the biophysical properties of the GluD1_R_-channel are like other members of the ionotropic glutamate receptor family. Given the widespread distribution of these receptors throughout the brain (Hepp et al., 2015), ion channel function of GluD1_R_ may be prevalent and relevant to neuronal excitability and circuit function in different parts of the throughout the nervous system. This study lays the foundation to investigate the ion channel function of GluD1_R_ in excitatory G_q_PCR-dependent synaptic transmission and regulation of neuronal excitability, expanding upon the wealth of knowledge of pharmacology and regulatory elements established for NMDA_R_ and AMPA_R_ signaling.

## Materials and methods

**Key resources table:** 

Reagent type (species) or resource	Designation	Source or reference	Identifiers	Additional information
Strain, strain background (*Mus musculus*)	C57BL/6J	The Jackson Laboratory	Stock# 000664 RRID:IMSR_JAX:000664	males and females
Strain, strain background (*E. coli*)	NEB Stable	New England Biolabs	Cat# C3040H	-
Genetic reagent (adeno-associated virus)	AAV-Cas9	PMIDs:25326897 30792150	NIDA IRP Core Facility, AAV1, pX551, RRID:Addgene_60957	Lot# AAV692
Genetic reagent (adeno-associated virus)	AAV-*Grid1*	This paper	NIDA IRP Core Facility, AAV1, pOTTC1706, Addgene 131683	Lot# AAV732
Genetic reagent (adeno-associated virus)	AAV-empty	This paper	NIDA IRP Core Facility, AAV1, pOTTC1553, Addgene 131682	Lot# AAV746
Chemical compound, drug	Bgl I restriction enzyme	New England Biolabs	Cat# R0143S	-
Chemical compound, drug	D-serine	Millipore Sigma	Cat# S4250	10 mM
Chemical compound, drug	GDPβS-Li_3_	Millipore Sigma	Cat# G7637	1.8 mM
Chemical compound, drug	Glycine	Millipore Sigma	Cat# G7126	10 mM
Chemical compound, drug	Idazoxan	Millipore Sigma	Cat# I6138	1 μM
Chemical compound, drug	Kynurenic acid	Millipore Sigma	Cat# K3375	1 mM
Chemical compound, drug	MK-801	Tocris Bioscience	Cat #0924	5 μM
Chemical compound, drug	NASPM	Tocris Bioscience	Cat #2766	100 μM
Chemical compound, drug	NBQX	Tocris Bioscience	Cat #1044	3 μM
Chemical compound, drug	NMDG	Millipore Sigma	Cat# 66930	126 mM
Chemical compound, drug	Noradrenaline	Tocris Bioscience	Cat #5169	30 μM
Chemical compound, drug	Picrotoxin	Tocris Bioscience	Cat #1128	100 μM
Chemical compound, drug	Prazosin	Millipore Sigma	Cat# P7791	100 nM
Chemical compound, drug	Reserpine	Millipore Sigma	Cat# R0875	1 μM
Chemical compound, drug	Strychnine	Millipore Sigma	Cat# S8753	10 μM
Chemical compound, drug	Tetrodotoxin	Tocris Bioscience	Cat# 1069	1 μM
Chemical compound, drug	WAY-100635	Tocris Bioscience	Cat# 4380	300 nM
Sequence-based reagent	Forward amplification primer	IDTDNA	tgattacgccaagctt GGTGGAGCTGTGTGGATGAAGC	-
Sequence-based reagent	Forward sequence primer	IDTDNA	CCAGCCTGTGACCTCATGACC	-
Sequence-based reagent	Reverse amplification primer	IDTDNA	gacggccagtgaattc CTTCAGCTGTCATGATAAGGTGATGTTG	-
Commercial assay, kit	In-Fusion HD Cloning	Takara Bio Clontech	Cat# 639647	-
Software, algorithm	Clampfit 10.7	Axon Instruments	RRID:SCR_011323	https://www.moleculardevices.com/products/axon-patch-clamp-system
Software, algorithm	EthoVision XT	Noldus Information Technology	RRID:SCR_000441	https://www.noldus.com/ethovision-xt
Software, algorithm	Fiji	PMID:22743772	RRID:SCR_002285	http://fiji.sc
Software, algorithm	Prism 8	GraphPad	RRID:SCR_002798	http://www.graphpad.com
Software, algorithm	VersaMax Analyzer	Omnitech-electronics, Inc	http://www.omnitech-electronics.com/product/VersaMax-Legacy-Open-Field---Locomotor-Activity/1930	-

### Animals

All studies were conducted in accordance with the National Institutes of Health Guide for the Care and Use of Laboratory animals with the approval of the National Institute on Drug Abuse Animal Care and Use Committee. Wild-type C57BL/6J (>3 months old) mice of either sex were used. Mice were group-housed on a 12:12 hr reverse light cycle.

### Brain slice preparation and electrophysiological recordings

The methods for brain slice preparation and electrophysiological recordings were almost identical to previous reports in the dorsal raphe (Gantz et al., 2015a) and ventral midbrain (Gantz et al., 2015b). In brief, mice were deeply anesthetized with isoflurane and killed by decapitation. Brains were removed quickly and placed in warmed artificial cerebral spinal fluid (modified Krebs’ buffer) containing (in mM): 126 NaCl, 2.5 KCl, 1.2 MgCl_2_, 1.2 CaCl_2_, 1.2 NaH_2_PO_4_, 21.5 NaHCO_3_, and 11 D-glucose with 5 μM MK-801 to reduce excitotoxicity and increase viability, bubbled with 95/5% O_2_/CO_2_. In the same solution, coronal dorsal raphe slices (220 μm) were obtained using a vibrating microtome (Leica 1220S) and incubated at 32°C > 30 min prior to recording.

Slices were then mounted in a recording chamber and perfused ~3 mL/min with ~35°C modified Krebs’ buffer. Electrophysiological recordings were made with a Multiclamp 700B amplifier (Molecular Devices), Digidata 1440A A/D converter (Molecular Devices), and Clampex 10.4 software (Molecular Devices) with borosilicate glass electrodes (King Precision Glass) wrapped with Parafilm to reduce pipette capacitance (Gantz and Bean, 2017). Pipette resistances were 1.8–2.8 MΩ when filled with an internal solution containing, (in mM) 104.56 K-methylsulfate, 5.30 NaCl, 4.06 MgCl_2_, 4.06 CaCl_2_, 7.07 HEPES (K), 3.25 BAPTA(K4), 0.26 GTP (sodium salt), 4.87 ATP (sodium salt), 4.59 creatine phosphate (sodium salt), pH 7.32 with KOH, mOsm ~285, for whole-cell patch-clamp recordings. Series resistance was monitored throughout the experiment. Transmitter release was evoked by trains of electrical stimuli delivered via a Krebs’ buffer-filled monopolar stimulating electrode positioned in the dorsal raphe, within 200 μm of the recorded neuron (Gantz et al., 2015a). Cell-attached recordings were made from quiescent neurons in slice, using pipettes filled with modified Krebs’ buffer. For experiments involving viral microinjections, transduced neurons were identified in the slice by visualization of eGFP. Reported voltages are corrected for a liquid junction potential of −8 mV between the internal solution and external solution. All drugs were applied by bath application. All experiments were conducted following incubation in an NMDA_R_ channel blocker (MK-801, 5 μM,>1 hr), and then with AMPA_R_ and Kainate_R_ (NBQX, 3 μM), GABA-A_R_ (picrotoxin, 100 μM), and 5-HT1A_R_ (WAY-100635, 300 nM) antagonists in the external solution. In addition, a α2-adrenergic receptor antagonist (idazoxan, 1 μM) was added for experiments where noradrenaline was applied and a glycine receptor antagonist (strychnine, 10 μM) was added when glycine was applied. Unitary current was calculated from fluctuation analysis, as previously described (Bean et al., 1990), assuming the macroscopic current arises from independent, identical channels with a low probability of opening, according probability theory; *i* = σ^2^/[*I*(1 p)] where *i* is unitary current, σ^2^ is the variance, *I* is mean current amplitude, and *p* is probability of opening.

### Vector construction gRNA identification

CRISPR SpCas9 gRNA target sites were identified in the mouse *Grid1* gene (NC_000080.6) using CRISPOR (Haeussler et al., 2016). The seed sequence (GAACCCTAGCCCTGACGGCG) was chosen based on its relatively high specificity scores and the observation that it contains a Bgl I restriction enzyme site (GCCNNNN^NGGC) that overlaps with the Cas9 cleavage site.

### Mouse *Grid1* genotyping

C57BL/6J mouse genomic DNA was isolated from tail biopsies or brain pieces containing microdissected dorsal raphe by digestion in DNA lysis buffer (50 mM KCl, 50 mM Tris-HCl (pH 8.0), 2.5 mM EDTA, 0.45% NP-40, 0.45% Tween-20, 0.5 ug/mL proteinase K) for 3 hr at 55°C, and 1 hr at 65°C. Lysates were then used as templates to amplify a 654 basepair fragment including the 390F gRNA target site using Q5 HotStart Master mix (New England Biolabs). A portion of the finished PCR reaction was treated with Bgl I restriction enzyme (New England Biolabs) for 60 min and processed on an AATI fragment analyzer.

### Construction and packaging of AAV vectors

The AAV vector plasmid encoding SpCas9 (Swiech et al., 2015) (pX551) expressed from the *Mecp2* promoter was a gift from Feng Zhang (Addgene plasmid # 60957, AAV-Cas9). The AAV packaging plasmid encoding a nuclear envelope-embedded eGFP reporter (Addgene 131682) was constructed by amplifying the KASH domain from (Addgene 60231, a gift of Feng Zhang) and fusing it (in-frame) to the end of coding region for eGFP in (Addgene 60058, pOTTC407) using ligation-independent cloning (AAV-empty, Figure 5—figure supplement 1A). gRNA was cloned into a mU6 expression cassette and then moved into an AAV backbone expressing a nuclear envelope-embedded (KASH-tagged) eGFP reporter (Addgene 131683) by PCR amplification and ligation-independent cloning (AAV-*Grid1*). Insert-containing clones were verified by sequencing and restriction fragment analysis prior to virus production. All AAV vectors were produced using triple transfection method as previously described (Howard and Harvey, 2017). All vectors were produced using serotype 1 capsid proteins and titered by droplet digital PCR.

### Stereotaxic intracranial microinjections

Mice were anesthetized with a cocktail of ketamine/xylazine, immobilized in a stereotaxic frame (David Kopf Instruments), and received one midline injection of a 1:1 (v/v) cocktail of viruses AAV-Cas9 and AAV-empty or AAV-*Grid1* for total volume 400 nL delivered over 4 min. The coordinates for injection were AP −4.4; ML 1.19, 20° angle; DV −3.62 mm, with respect to bregma. Prior to surgery, mice were injected subcutaneously with warm saline (0.5 mL) to replace fluid lost during surgery and given carprofen (5 mg/kg) post-surgery for pain relief. Mice recovered for >4 weeks to allow expression.

### Behavioral assays

Behavioral assays were conducted during the dark cycle, using 3 separate cohorts of AAV-Cas9 and AAV-empty or AAV-*Grid1*-injected mice as biological replicates, 30–55 d post-injection. To measure basal locomotion, mice were placed in locomotor boxes (VersaMax System, Omnitech Electronics, Inc) in a dark room for 1 hr, following prior habituation to the locomotor boxes for >2 d (1 hr/d). VersaMax Analyzer software was used to determine the total distance traveled, time spent moving, and velocity of movement in the last 30 min of the session. The boxes were cleaned with 70% ethanol and allowed to dry between trials. The elevated-plus maze was used to assay anxiety-related behaviors (Walf and Frye, 2007). The apparatus (Med associates, Inc) was placed 30 cm above the floor and consisted of two plastic light gray open arms (30 × 5 cm) and two black enclosed arms (30 × 5 cm) extending from a central platform (5 × 5 × 5 cm) at 90 degrees. Following habituation to the brightly lit room, mice were placed individually in the center of the maze, facing an open arm. Video tracking EthoVision XT software (Noldus Information Technology) was used to track mouse location, total distance traveled, velocity of movement, body elongation, and entries and time spent into the open and enclosed arms for each 5 min trial. Duration of head-dips, grooming, and enclosed-arm rearing were scored manually from videos played a 0.5x speed. Rearing in the enclosed arm was often associated with pressing one or both forepaws to the wall. Stretched-attend postures was defined by body elongation (70% threshold) and movement velocity <1 cm/s. No mice fell or jumped from the maze and open-arm rearing was not observed. The maze was cleaned with 70% ethanol after every trial and allowed to dry before the next trial. Mice were excluded from analysis if there was limited or no expression in the dorsal raphe, or if expression spread rostrally to the ventral tegmental area or caudally to locus coeruleus.

### Immunohistochemistry and confocal microscopy

Following behavioral assays, mice were euthanized with Euthasol and transcardially perfused with PBS followed by ice-cold 4% paraformaldehyde in PBS (pH 7.4). Brains were fixed overnight at 4 C and then sliced coronally in 50 μm sections. Alternatively, mice were anesthetized with isoflurane and euthanized by decapitation. Brains were removed and slices were prepared as for brain slice electrophysiology (220 μm), then fixed in room temperature 4% paraformaldehyde in PBS for 1 hr. Slices were mounted with Fluoromount-G with DAPI (Invitrogen) aqueous mounting medium. Confocal images were collected on an Olympus microscope (4x, 0.16 NA) and processed using Fiji.

### Data analysis and visualization

Data were analyzed using Clampfit 10.7. Data are presented as representative traces, or in scatter plots where each point is an individual cell, and bar graphs with means ± SEM. In traces with electrical stimulation, stimulation artifacts have been blanked for clarity. Unless otherwise noted, *n* = number of distinct cells or mice as biological replicates. No sample was tested in the same experiment more than once (technical replication). E_rev_s were determined by linear regression for each cell. Recordings in which current did not cross 0 pA were omitted from analysis. To minimize space-clamp errors, analysis of current during voltage ramps was limited to −10 mV where the currents were typically less than 500 pA. Ramp currents were averaged in 2 mV bins (20 ms). Data sets with n > 30 were tested for normality with a Shapiro-Wilk test. When possible (within-group comparisons), significant differences were determined for two group comparisons by paired t-tests, Wilcoxon matched-pairs signed rank test, and in more than two group comparisons by nonparametric repeated-measures ANOVA (Friedman test). Significant mean differences in between-group comparisons were determined for two group comparisons by Mann Whitney tests, and in more than two group comparisons by Kruskal-Wallis tests. ANOVAs were followed, when p<0.05 by Dunn’s multiple comparisons post hoc test. Linear trends were analyzed using a mixed model ANOVA. A difference of p<0.05 was considered significant. For behavioral assays, Grubbs test was used to identify outliers. Basal locomotion and time spent in stretched-attend posture from one *Grid1* mouse each were found to be outliers and were excluded from group comparisons. Exact values are reported unless p<0.0001 or>0.999. Statistical analysis was performed using GraphPad Prism 8 (GraphPad Software, Inc).

## Data Availability

All data analysed for this study are included in the manuscript.

## Additional files

Transparent reporting form
