# Supplementary material for: Delta glutamate receptor conductance drives excitation of mouse dorsal raphe neurons
Source: eLife. 2020 Apr 1;9:e56054. doi: 10.7554/eLife.56054 (PMC7180053; doi:10.7554/eLife.56054)
Supplement: Peer review history

# Decision letter

**DOI:** 10.7554/eLife.56054.sa1

**Affiliations:** Aix-Marseille University, INSERM, INMEDFrance; Aix-Marseille University, INSERM, INMEDFrance; University of WashingtonUnited States; University of California, San FranciscoUnited States

In the interests of transparency, eLife publishes the most substantive revision requests and the accompanying author responses.

**Acceptance summary:**

This study provides an elegant and convincing pharmacological and biophysical demonstration that the orphan δ glutamate receptor is responsible for α-Adrenergic receptor-mediated depolarization of dorsal raphe neurons. Genetic tools reveal how a nanoscale alteration (cell type specific deletion of GluD in in the dorsal raphe) leads to macroscale behavioral disturbances.

**Decision letter after peer review:**

Thank you for submitting your article "δ glutamate receptor conductance drives excitation of dorsal raphe neurons" for consideration by *eLife*. Your article has been reviewed by three peer reviewers, including Olivier J Manzoni as the Reviewing Editor and Reviewer #1, and the evaluation has been overseen by Gary Westbrook as the Senior Editor. The following individuals involved in review of your submission have agreed to reveal their identity: Bertil Hille (Reviewer #2); Kevin J Bender (Reviewer #3). The reviewers have discussed the reviews with one another and the Reviewing Editor has drafted this decision to help you prepare a revised submission.

This study provides an elegant and convincing pharmacological and biophysical demonstration that the orphan δ glutamate receptor is responsible for α-Adrenergic receptor-mediated depolarization of dorsal raphe neurons. Astute genetic tools are used to show how a nanoscale alteration (cell type specific deletion of GluD in in the dorsal raphe) leads to macroscale disturbances (anxiogenic behaviors).

Essential revisions:

1) Improve the form of the manuscript (no experiments required; analysis and rewriting required):

1A) Abstract

The Abstract summarizes the principal point of the paper with "We show that a1-AR-mediated excitatory synaptic transmission is mediated by δ glutamate receptors (GluD1_R_)." To paraphrase, this sounds like "The response mediated by receptor X is mediated by receptor Y," This sentence and possibly others like it do not do justice to the paper and should be improved. Clarify that the main and important discovery is that the a1-AR-mediated postsynaptic depolarization of raphe neurons results from the G-protein coupled modulation of a non-selective cation channel. This channel happens to be named δ glutamate receptor (GluD1_R_), but that may be a distracting misnomer. It may not be glutamate responsive, and is, so far, an orphan ionotropic receptor homolog that may not act as a receptor at all. It is an ion channel at least.

1B) Materials and methods:

– You say, "With cell-attached recordings, a train of 5 electrical stimuli (60 Hz) produced firing in previously quiescent neurons."

Because this stimulation was key in demonstrating physiological effects it is important to clearly explain where or how the cell is stimulated (was a stimulus applied extracellularly to some other part of the slice?).

– The part about the preparation is written without references or detail as if everyone would know what was done already. Indeed, the whole Materials and methods section makes little reference to the literature except for a reference to noise analysis and one to how to generate AAV virus tools.

– Concentrations for the antagonists should be mentioned.

1C) Figures:

– Adding color in the figures would help readers.

– Some of the supplementary figures could easily be incorporated into the main figures, as they are important controls. For example, why not include Figure 1—figure supplement 1 in the main figure? The same stands for Figure 2—figure supplements 1 and 2. Figure 6—figure supplements 1 and 2 could be combined to 1 package (remaining as a supplement).

1D) Results:

– You write "We applied the channel selective Joro spider toxin, 1-Naphthyl acetyl spermine (NASPM), which is an open-channel blocker GluD_R_, akin to other Ca^2+^-permeable ionotropic glutamate receptors (Benamer et al., 2018; Blaschke et al., 1993; Guzmán et al., 2017; Kohda et al., 2000).”

Please rephrase: We applied 1-Naphthyl acetyl spermine (NASPM), a synthetic analogue of Joro spider toxin that is an open-channel blocker of some other Ca^2+^-permeable ionotropic glutamate receptors and of GluD_R_ channels (Koike, Iino and Ozawa, 1997).

– You write, "GDP-bS-Li3."

GDPbS-Li3 would be a more familiar name for this compound.

– The behavioral analysis is very limited, especially in comparison with the depth of the biophysical data. At the very least the authors should report rears, head dips, freezing and stretched-attend postures, from their EPM experiments (i.e. already been performed).

1E) Discussion:

– The reversal potential of -20 mV suggests that the channel would be twice as permeable to K as to Na--if it really is the response from one channel type in a space-clamped cell. In the Discussion however the authors draw back from being sure that it is one channel and that there is space clamp.

– The signal from the adrenergic receptor to the depolarizing channel is not yet specified. The MS stimulates mechanistic questions on intracellular signaling that I hope will be addressed in future work, including: Would other Gq-coupled receptors activate this channel? Can the phenomena be reproduced in expression systems? Is GluD_R_ activated via any of the following traditional α-1 signaling pathways: IP3-Ca, DAG-PKC, PIP2 depletion, or arrestin-Mek-Erk? Could any of them explain why the epsc response has such a long latency and such long persistence? These points could be added to the Discussion.

– What explains the incomplete blockade by glycine+strychnine, D-Serine and Kynurenate? Additional a1-AR mediated but not GluD_R_-mediated mechanisms (although the elimination of a1-AR-EPSC by targeting of GluD1 receptors via CRISPR/Cas9 argues against this idea)? Non saturating doses of antagonists?

2) Clarify and expand the current data set (additional experiments may be required):

2A) Figure 1:

It would be great to see the contribution of GluD1_R_ compared to traditional ionotropic signaling in Figure 1A. In other words, an example of stimulation in the absence of the AMPA/NMDA/GABA-A/5-HT1A antagonist cocktail, chased with the cocktail to then show the remaining GluD1_R_ mediated synaptic event.

2B) Figure 7:

If animals are available, the authors may consider 1/ testing the (acute) effects of SSRIs on the reported anxiogenic phenotype and/or 2/ evaluating how the lack of functional GluD1 receptors in the dorsal raphe impact social interaction and social memory (both likely to reflect the elevated anxiety reported here). If time was not a concern, one may also evaluate how knocking out GluD-a1-AR impacts on the susceptibility to stress and stress-induced depressive behaviors.

# Reply: Author response

**DOI:** 10.7554/eLife.56054.sa2

Essential revisions:

1) Improve the form of the manuscript (no experiments required; analysis and rewriting required):

1A) Abstract

The Abstract summarizes the principal point of the paper with "We show that a1-AR-mediated excitatory synaptic transmission is mediated by δ glutamate receptors (GluD1_R_)." To paraphrase, this sounds like "The response mediated by receptor X is mediated by receptor Y," This sentence and possibly others like it do not do justice to the paper and should be improved. Clarify that the main and important discovery is that the a1-AR-mediated postsynaptic depolarization of raphe neurons results from the G-protein coupled modulation of a non-selective cation channel. This channel happens to be named δ glutamate receptor (GluD1_R_), but that may be a distracting misnomer. It may not be glutamate responsive, and is, so far, an orphan ionotropic receptor homolog that may not act as a receptor at all. It is an ion channel at least.

This is an excellent point. As suggested, we have changed the Abstract to emphasize modulation of a non-selective cation channel. Through-out the text, we now refer to the ion channel as the “δ glutamate receptor-channel” or “GluD1_R_ channel”, and “ionotropic function” has been changed to “ion channel function”. Lastly, we have stated in the Discussion that our study shows that GluD1_R_ acts as an ion channel, but not necessarily a ligand-gated ionotropic receptor, in so far as the identify of a chemical messenger or ligand responsible for activation, if it exists, remains unknown.

1B) Materials and methods:

– You say, "With cell-attached recordings, a train of 5 electrical stimuli (60 Hz) produced firing in previously quiescent neurons."

Because this stimulation was key in demonstrating physiological effects it is important to clearly explain where or how the cell is stimulated (was a stimulus applied extracellularly to some other part of the slice?).

Yes, all stimuli were applied via a monopolar electrode placed in the brain slice within 200 microns to the recorded neurons. We have now included more details on the methodology in the Results and Materials and methods sections, as well as reference prior studies with similar methodology.

– The part about the preparation is written without references or detail as if everyone would know what was done already. Indeed, the whole Materials and methods section make little reference to the literature except for a reference to noise analysis and one to how to generate AAV virus tools.

More details and references have now been added to the Materials and methods section. We thank the reviewers for bringing this to our attention.

– Concentrations for the antagonists should be mentioned.

These are now included in the new revised manuscript.

1C) Figures:

– Adding color in the figures would help readers.

We have added color to the new Figures 2, 5, and 6 to help readability.

– Some of the supplementary figures could easily be incorporated into the main figures, as they are important controls. For example, why not include Figure 1—figure supplement 1 in the main figure?

Figure 1—figure supplement 1 is now included in Figure 1.

The same stands for Figure 2—figure supplements 1 and 2.

Figure 2—figure supplement 1, Figure 2—figure supplement 2, and Figure 3 are now included in Figure 2. Subsequent figures numbers have adjusted to reflect the combination of Figure 2 and Figure 3.

Figure 6—figure supplements 1 and 2 could be combined to 1 package (remaining as a supplement).

Figure 6—figure supplements 1 and 2 are now combined into one supplementary figure (Figure 5—figure supplement 1).

1D) Results:

– You write "We applied the channel selective Joro spider toxin, 1-Naphthyl acetyl spermine (NASPM), which is an open-channel blocker GluD_R_, akin to other Ca^2+^-permeable ionotropic glutamate receptors (Benamer et al., 2018; Blaschke et al., 1993; Guzmán et al., 2017; Kohda et al., 2000).”

Please rephrase: We applied 1-Naphthyl acetyl spermine (NASPM), a synthetic analogue of Joro spider toxin that is an open-channel blocker of some other Ca^2+^-permeable ionotropic glutamate receptors and of GluD_R_ channels (Koike, Iino and Ozawa, 1997).

This has been rephrased with the suggested reference.

– You write, "GDP-bS-Li3."

GDPbS-Li3 would be a more familiar name for this compound.

This is corrected in the revised manuscript.

– The behavioral analysis is very limited, especially in comparison with the depth of the biophysical data. At the very least the authors should report rears, head dips, freezing and stretched-attend postures, from their EPM experiments (i.e. already been performed).

We have now included time spent grooming, head-dipping off the open arms, rearing in the enclosed arms, and stretched-attend postures. The data show a selective deficit in exploratory behavior in the open arms (head-dipping) following GRID1 deletion while rearing in the enclosed arms was similar to control mice. We thank the reviewers for this suggestion. The new data advances the understanding of the phenotype.

1E) Discussion:

– The reversal potential of -20 mV suggests that the channel would be twice as permeable to K as to Na--if it really is the response from one channel type in a space-clamped cell. In the Discussion however the authors draw back from being sure that it is one channel and that there is space clamp.

We have extensively revised this Discussion paragraph. By our calculations using a reversal potential of -30 mV, and the shift in reversal potentials observed with changing extracellular potassium, we estimate the channel may be 2 to 3 times as permeable to potassium as sodium, now included in the Results.

Regarding the issue of space-clamp error, we feel it is important to acknowledge that recordings in brain slices can particularly susceptible to some space-clamp error, without careful consideration. In our study, all efforts were made to reduce space-clamp errors, including the use of slow voltage ramps (1 mV/10 mS) where data were binned every 20 ms. Voltage ramps were made from -120 to -10 mV where the size of the total membrane current was less than 500 pA. Further, we also used voltage steps (10 mV increments, 150 ms duration) to generate current-voltage plots of the current induced by exogenous noradrenaline, and obtained identical E_rev_s (data not shown in the manuscript). Thus, we do not believe that space-clamp error accounts for the difference in reports of reversal potential of constitutively open mutant GluD_R_ channels (0 mV) and our data (~ -30 mV). These points are clarified in the revised text.

Regarding the issue of the one channel, the results following functional deletion of GluD1_R_ indicate that the α1-A_R_-dependent *inward* current is carried completely by GluD1_R_ channels. We have removed reference to inhibition of A-type potassium current by α1-A_R_ activation (Aghajanian, 1985). While an important finding, the data from our study do not support involvement of A-type channels in the α1-A_R_-EPSC.

– The signal from the adrenergic receptor to the depolarizing channel is not yet specified. The MS stimulates mechanistic questions on intracellular signaling that I hope will be addressed in future work, including: Would other Gq-coupled receptors activate this channel? Can the phenomena be reproduced in expression systems? Is GluD_R_ activated via any of the following traditional α-1 signaling pathways: IP3-Ca, DAG-PKC, PIP2 depletion, or arrestin-Mek-Erk? Could any of them explain why the epsc response has such a long latency and such long persistence?

These points could be added to the Discussion.

We have revised this part of the Discussion to include these points. In brief, it remains to be determined whether other GqPCRs augment GluD1_R_ channel current. Brown et al., 2002 (cited in the manuscript) find that the α1-A_R_-dependent inward current occludes an inward current produced by activation of Gq-coupled histamine and orexin receptors. This suggests that these receptors may also be modulating GluD1_R_ channels, but it has not yet been tested.

In regard to expression systems, Benamer et al., 2018, were able to measure mGluR-GluD1_R_ current in HEK cells suggesting we may be able to reproduce our α1-A_R_ phenomena in expression systems. Instead of expression systems, we will be working towards recordings from acutely dissociated dorsal raphe neurons to see if the tonic current requires brain slice work. This model will allow for better voltage control and high-throughput screening of drugs and then extend our findings to expression systems.

In regard to the mechanism, the inclusion of intracellular BAPTA in the recording solution makes it unlikely that α1-A_R_s are modulating GluD1_R_ channels via IP_3_-Ca^2+^. Involvement of PIP2 hydrolysis to DAG, and the subsequent signaling cascade is a very viable possibility. The duration of the α1-A_R_s (on average 27s, but can persist up to 90s) is similar to time course of recovery (tens-of-seconds to minutes) of M-current inhibition following PIP2 hydrolysis and inhibition of A-type potassium current by 2-AG (citations in manuscript). Alternatively, there may be direct modulation by G protein subunits, activation of protein kinase signaling cascades (e.g. Arrestin-MEK-ERK, or PKC), or a combination of effectors, akin to GIRK channel gating by Gβγ subunits and PIP2. We feel to present the results comprehensively, they are best fleshed out in a full follow-up study. But these possibilities are now discussed in the revised manuscript.

– What explains the incomplete blockade by glycine+strychnine, D-Serine and Kynurenate? Additional a1-AR mediated but not GluD_R_-mediated mechanisms (although the elimination of a1-AR-EPSC by targeting of GluD1 receptors via CRISPR/Cas9 argues against this idea)? Non saturating doses of antagonists?

This is a great question. In other publications, there is never complete elimination of GluD_R_ channel current at concentrations up to 10 mM. D-serine or glycine reduce GluD_R_ channel current, with reports of near complete reduction of the *Lurcher* GluD2_R_ mutant (~25% of the current remains, e.g. Naur et al., 2007) and wild type GluD2_R_ (~35% of current remains, Ady et al., 2013) but partial reduction of mutant GluD1_R_ (~50-60% of the current remains, Yadav et al., 2011) and wild type GluD1_R_ (60% of the current remains, Benamer et al., 2018). The reductions we observe are consistent with these GluD1_R_ reports.

What explains incomplete blockade at a structural level is not completely understood. Hansen et al., 2009 (now referenced in the revised manuscript) examined the structural basis for reduction of constitutively open *Lurcher* GluD2_R_ channel current. Their conclusion is that binding of D-serine puts Lurcher GluD2_R_ in a closed but desensitized state. No similar study has been performed yet for GluD1_R_. In the revised manuscript, we have added new data examining membrane noise during the EPSC in control conditions and after reduction by D-serine or glycine. We report that there was no change in unitary channel current (Figure 4B and D) with D-serine nor glycine. The simplest explanation is that there is a change in the number of open channels, but further work will be needed to determine the structural basis for reduction. To make this clearer, we have revised the manuscript. Instead of stating these amino acids “inhibit” GluD_R_ channel current, we write that they “partially reduce” GluD_R_ channel current and reference these studies.

Our manuscript is the first to report reductions in GluD1_R_ channel-current by kynurenic acid. We discovered this serendipitously after initially using kynurenic acid to inhibit NMDA_R_ channel synaptic currents. This may be unique to GluD1_R_ channel current over GluD2_R_, as Kristensen et al. (2016, Mol Pharm) reported that Lurcher GluD2_R_ channel current is not sensitive to kynurenic acid but is reduced by the kynurenic acid analog, 7-chlorokynurenic acid (7-CKA). We feel it is best for reproducibility to report the effect of kynurenic acid since it is used commonly to inhibit NMDA_R_ synaptic responses. Then as a follow-up study, we will screen other known compounds and generate concentration-response curves as in Kristensen et al., 2016.

2) Clarify and expand the current data set (additional experiments may be required):

2A) Figure 1:

It would be great to see the contribution of GluD1_R_ compared to traditional ionotropic signaling in Figure 1A. In other words, an example of stimulation in the absence of the AMPA/NMDA/GABA-A/5-HT1A antagonist cocktail, chased with the cocktail to then show the remaining GluD1_R_ mediated synaptic event.

The revised manuscript now includes contribution of GluD1_R_ channel synaptic current in comparison to fast synaptic transmission and slow 5-HT1A_R_ synaptic transmission (Figure 1H). In this experiment, NMDA_R_ channels were blocked by MK-801, but contribution from NMDA_R_ channels at holding potential of -65 mV is expected to be minimal due to magnesium block. Otherwise, the experimental design is as suggested by the reviewers. We stimulated in the absence of AMPA/GABA-A/5-HT1A antagonists, then chased with a cocktail of antagonists to reveal only the GluD1_R_ channel-mediated current. We hope the addition of this figure helps illustrate the duration of the GluD1_R_ channel-mediated synaptic current.

2B) Figure 7:

If animals are available, the authors may consider 1/ testing the (acute) effects of SSRIs on the reported anxiogenic phenotype and/or 2/ evaluating how the lack of functional GluD1 receptors in the dorsal raphe impact social interaction and social memory (both likely to reflect the elevated anxiety reported here). If time was not a concern, one may also evaluate how knocking out GluD-a1-AR impacts on the susceptibility to stress and stress-induced depressive behaviors.

We are also very interested in extending the behavioral phenotype to examine other social and stress-induced behaviors. It is a great suggestion to test the effects of SSRIs. However, we feel it is beyond the scope of the present study. These questions will be addressed more completely in a study where we also evaluate the impact of the lack of functional GluD1_R_ in the dorsal raphe on serotonin release and serotonin receptor-dependent signaling.
